# The co-delivery of adenovirus-based immune checkpoint vaccine elicits a potent anti-tumor effect in renal carcinoma

**DOI:** 10.1038/s41541-023-00706-x

**Published:** 2023-08-04

**Authors:** Nan Jiang, Yanyan Zheng, Jiage Ding, Jiawei Wang, Fei Zhu, Meng Wang, Navid Sobhani, Praveen Neeli, Gang Wang, Hailong Li, Junnian Zheng, Dafei Chai

**Affiliations:** 1 Department of Urology, Suqian Hospital of Chinese Medicine Department of Pharmacy, Suqian, Jiangsu China; 2 Cancer Institute, Xuzhou Medical University, Xuzhou, Jiangsu China; 3 Center of Clinical Oncology, Affiliated Hospital of Xuzhou Medical University, Xuzhou, Jiangsu China; 4 Jiangsu Center for the Collaboration and Innovation of Cancer Biotherapy, Cancer Institute, Xuzhou Medical University, Xuzhou, Jiangsu China; 5 Department of Medicine, Baylor College of Medicine, Houston, TX USA; 6 Department of Urology, Affiliated Hospital of Xuzhou Medical University, Xuzhou, China

**Keywords:** Renal cell carcinoma, Preclinical research, DNA vaccines

## Abstract

Immune-based checkpoint therapy has made significant progress in cancer treatment, but its therapeutic effect is limited. A replication-defective adenovirus (Ad) vaccine encoding tumor antigen carbonic anhydrase IX (CAIX) combined with Ad-encoding immune checkpoint PD-L1 was developed to treat renal carcinoma. Three tumor models, subcutaneous, lung metastasis and orthotopic tumor were established, and Ad vaccines were used to immunize them and evaluate the vaccine’s therapeutic effect. Compared to the single Ad vaccine group, the subcutaneous tumor growth was significantly reduced in Ad-CAIX/Ad-PD-L1 combination group. Co-immunization of Ad-CAIX/Ad-PD-L1 enhanced the induction and maturation of CD11c^+^ or CD8^+^CD11c^+^ DCs in the spleen and tumor and promoted the strong tumor-specific CD8^+^ T cell immune responses. In vivo CD8 T cell deletion assay showed that the anti-tumor effect of the Ad-CAIX/Ad-PD-L1 vaccine was mainly dependent on functional CD8^+^ T cell immune responses. Furthermore, the Ad-CAIX/Ad-PD-L1 vaccine effectively inhibited tumor growth and lung metastasis in metastatic or orthotopic models. These results indicate that the combination strategy of the immune checkpoint vaccine shows promising potential as an approach for malignant tumor therapy.

## Introduction

Renal cell carcinoma (RCC) originating from renal epithelium, accounts for more than 90% of renal cancers^1,2^. There are three main histological subtypes of RCC: clear cell RCC (ccRCC), papillary RCC (pRCC), and chromophobe RCC (chRCC), with ccRCC being the most common subtype (around 75%)^2,3^. Unfortunately, metastatic ccRCC, due to its high expression of multidrug resistance protein 1 (MDR-1), doesn’t respond to conventional chemotherapy, resulting in increased mortality^1,4^. ccRCC is considered an immunogenic tumor because of the large number of infiltrating immune cells. Therefore, immunotherapy aimed at enhancing the immune responses of the immune system against tumor cells has become the focus, and it has continuously improved over the past decade^5^. Tyrosine kinase inhibitors, such as vascular endothelial growth factor (VEGF) receptor inhibitors, are used alone or in combination with immune checkpoint inhibitors (ICI) such as anti-programmed death 1 (PD-1), and have been approved for clinical application against many kinds of cancers^2^. These drugs are also promising in the treatment of ccRCC. Nevertheless, the therapeutic effects are limited, and many cases progress and eventually die^1,6^. Therefore, a new treatment strategy is urgently needed to overcome or eliminate the tumor.

DNA vaccine might be regarded as a promising immunotherapy strategy, inducing the immune system to trigger or enhance antigen-specific T cell responses and innate immune responses^7,8^. However, due to the poor immunogenicity of plasmid vectors, the application of DNA vaccines is limited^9^. Adenovirus (Ad) is a good choice because of its strong immunogenicity and high safety^10^. Ad vaccine administration has been shown to elicit robust antigen-specific CD8^+^ T cell immune responses against the coded antigens (Ag)^11^. Furthermore, in replication-deficient Ad, the essential viral gene E1 of their genome, is deleted through genetic modification and can be vectorized for easy manipulation^12–14^. Importantly, replication-deficient Ad vectors can also stimulate the expression of many transcripts encoding Ag by using strong exogenous promoters, such as the cytomegalovirus (CMV) promoter^15^. Studies have shown that Ad vaccines can effectively express Ag and induce the strong CD8^+^ T cell immune responses^16,17^ in both animal models^18,19^ and human^20^.

Choosing the most suitable tumor antigens, which are responsible for initiating tumor-specific immune responses, is incredibly important for the therapeutic efficacy of cancer immunity^8,11^. Carbonic anhydrase IX (CAIX), which is highly expressed at the tumor cell surface of ccRCC, participates in promoting the proliferation of tumor cells by maintaining cell pH regulation and is also considered as an attractive target of immunotherapy^21–23^. Studies have proved that monoclonal antibody (mAb) or DNA vaccines targeting CAIX exhibit a significant anti-tumor effect^24–26^. Recently, a phase 1 clinical study of dendritic cell (DC) vaccine based on CAIX has shown that patients with metastatic RCC confirmed a significant CAIX-specific immune response after three injection cycles of immunization^27^. In addition, our previous research has also demonstrated that DNA vaccines based on the combination of CAIX and an adjuvant can trigger antigen-specific CD8^+^ T cell immune responses in vivo^28,29^. However, using CAIX antigen alone is not sufficient to effectively induce anti-tumor T cell immunity^30,31^. Programmed death ligand-1 (PD-L1), also known as B7-H1 or CD274, is one of two main ligands for programmed death-1 (PD-1)^6^. The expression of PD-L1 can be observed in many tumors, such as lung carcinoma, melanoma, pancreatic carcinoma, and RCC^32^. According to a previous study, RCCs that overexpress PD-L1 are highly aggressive^33^. It has been proposed that the interaction between PD-1 and PD-L1 negatively regulates the immune responses, limits T-cell anti-tumor immunity, and promotes tumor immune evasion^34^. The results of the various experiments show that immunization with PD-L1 protein or DNA vaccines could produce effective anti-tumor immunity in different tumors^35–37^. Therefore, PD-L1 may enhance T-cell anti-tumor immune responses and inhibit tumor growth as an immune checkpoint vaccine.

In this study, we developed a replication-defective Ad vaccine encoding CAIX or PD-L1 antigen and evaluated its therapeutic effect in subcutaneous, orthotopic, or metastatic renal carcinoma models. Our results showed that the Ad-CAIX/Ad-PD-L1 vaccine significantly enhanced the induction and maturation of DCs or DC subsets, promoted the multifunctional CD8^+^ T-cell responses, and inhibited the growth of tumor growth in mice bearing hCAIX-Renca. The infiltration of CD8^+^ T cells and the production of TNF-α, IL-2, or IFN-γ cytokines on CD8^+^ T cells were increased in tumors. In metastatic or orthotopic tumor models, Ad-CAIX/Ad-PD-L1 vaccine significantly prevented lung metastasis and tumor growth. In conclusion, our research represents a novel strategy to treat malignant tumors by using the Ad-CAIX/Ad-PD-L1 vaccine.

## Results

### Ad-CAIX/Ad-PD-L1 inhibited the growth of the subcutaneous tumor and increased the induction of DCs and CD8^+^ T cells in the tumor

**Fig. 1:**
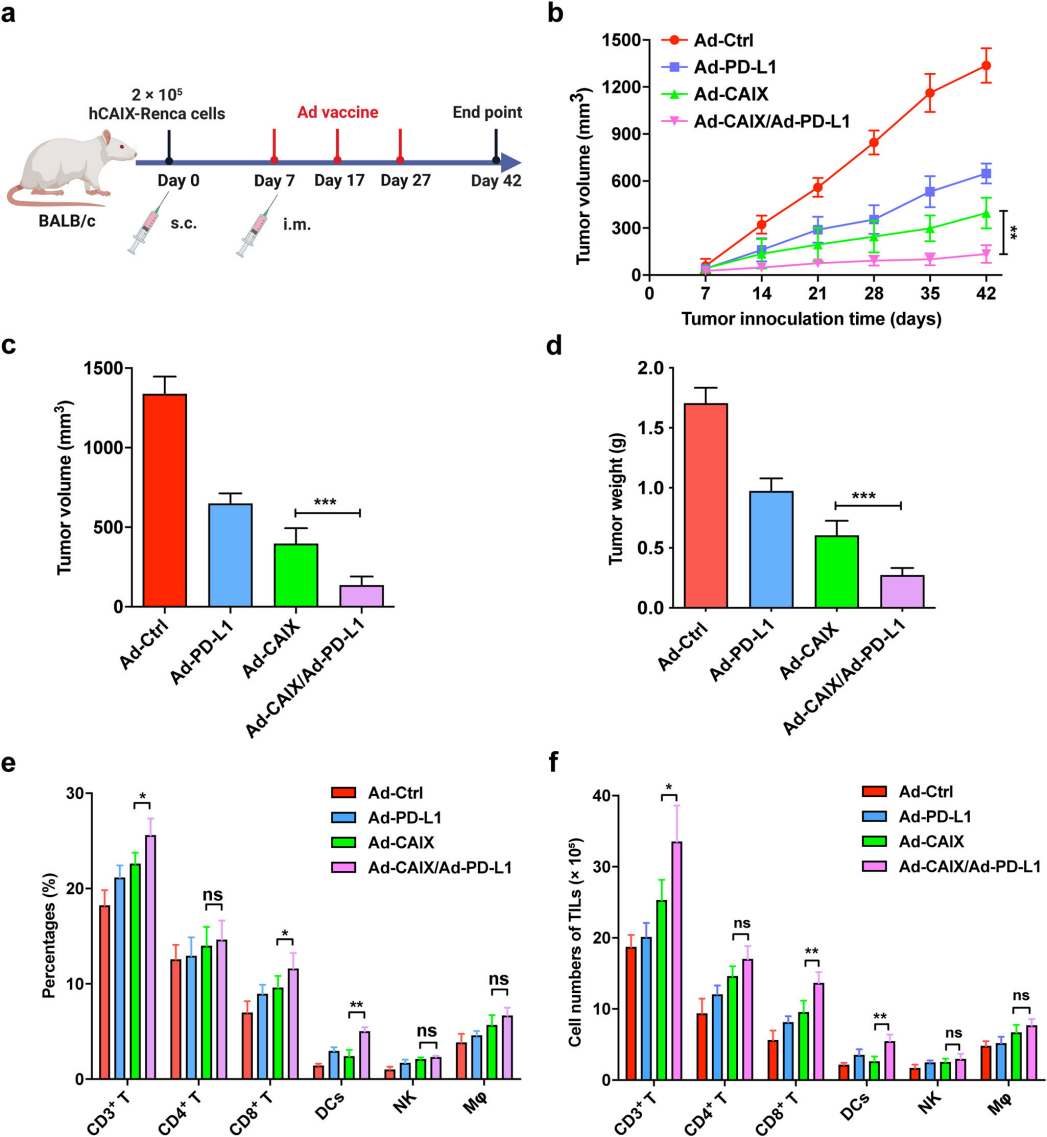
The therapeutic effect of Ad-CAIX/Ad-PD-L1 in the hCAIX-Renca subcutaneous tumor model. **a** Schematic diagram of the subcutaneous hCAIX-Renca tumor model by intramuscular injection of various vaccines on the 7th day after tumor inoculation. **b** The volume of subcutaneous tumors was measured weekly to evaluate tumor progression. **c** Tumor volumes of each group were measured 42 days post tumor inoculation. **d** Tumor weights of each group were measured at the experimental endpoint. **e**, **f** The percentages and total numbers of T cells, CD8^+^ T cells, CD4^+^ T cells, NK cells, DCs, and macrophages in TILs from tumors of immunized mice in each group. The data shown are the representative of three experiments. Data are shown as mean ± SD. The different significance was set at *p < 0.05, **p < 0.01, and ***p < 0.001; ns not significant. Multiple groups of comparison data were analyzed by one-way ANOVA.

To evaluate the therapeutic effect of Ad vaccines, Ad-Ctrl, Ad-CAIX, or Ad-PD-L1 were prepared. The results showed that these Ad vaccines could effectively express the encoded genes (Supplementary Fig. 1). Next, six-week-old BALB/c mice were used to establish subcutaneous tumor models, and 2 × 10^5^ hCAIX-Renca cells were inoculated to the right flank of the mice and then randomly grouped seven days later. On days 7, 17, and 27 post-tumor inoculation, the Ad vaccines were administered to the lateral thighs of the mice with a total dose of Ad-CAIX or Ad-PD-L1 of 9 × 10^8^ pfu per mouse (Fig. 1a). In order to evaluate the therapeutic efficacy of the vaccine Ad-CAIX/Ad-PD-L1, the growth of the tumor was continuously monitored. Compared with the Ad-CAIX-treated group, Ad-CAIX/Ad-PD-L1-treated group exhibited that the growth of subcutaneous tumors was inhibited (Fig. 1b), and the tumor volume was significantly reduced (Fig. 1c). Comparison of tumor weights in mice revealed significantly lower tumor weights in the Ad-CAIX/Ad-PD-L1 group than in the Ad-CAIX group (Fig. 1d). The above results indicated that combined immunization with vaccine Ad-CAIX/Ad-PD-L1 could significantly inhibit the growth of subcutaneous tumors in mice.

**Fig. 2:**
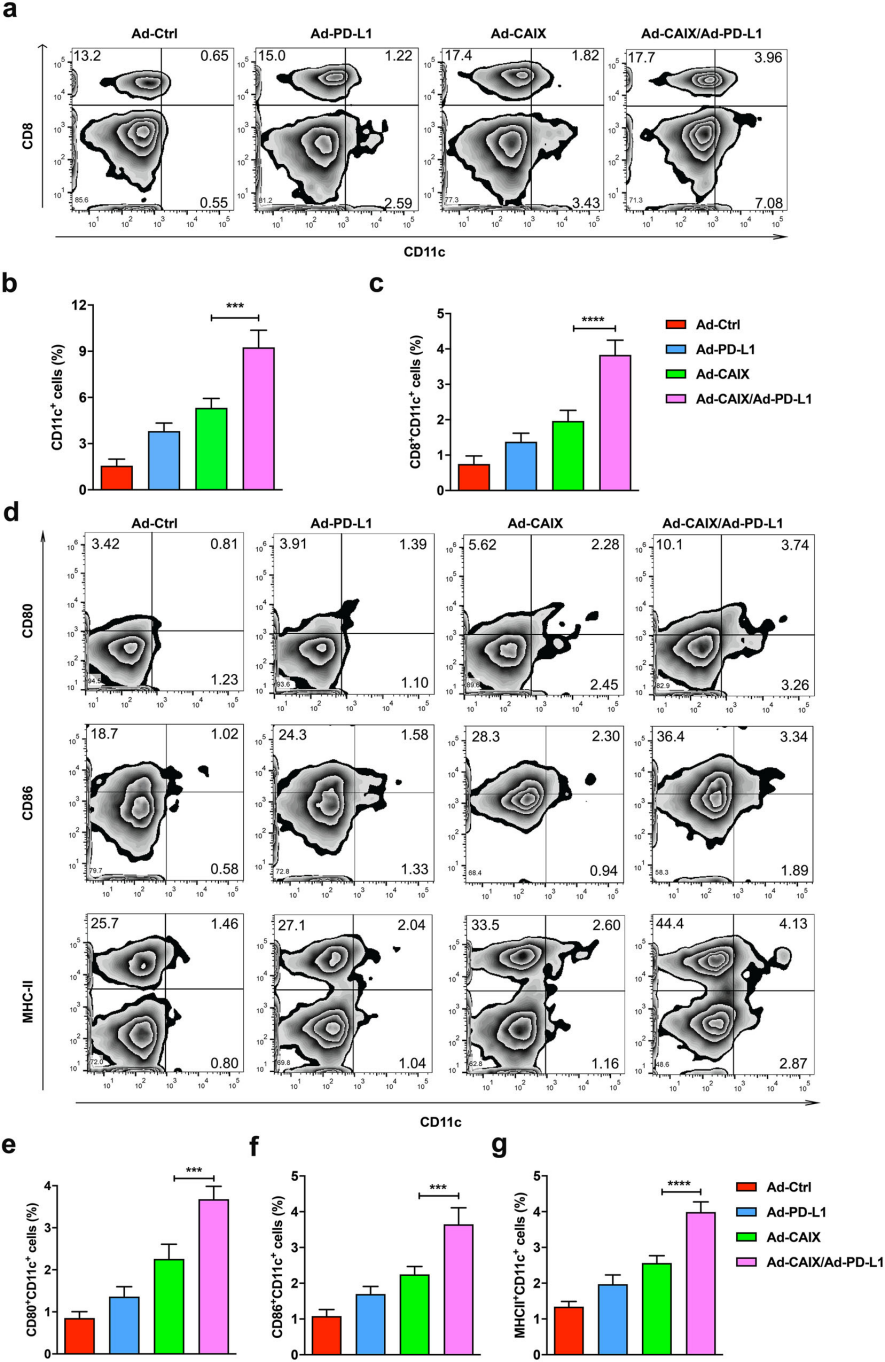
Ad-CAIX/Ad-PD-L1 promoted the induction and maturation of DCs in vivo. The induction of CD11c^+^ DCs or CD11c^+^CD8^+^ DCs in the spleens of mice immunized with different vaccines was analyzed by flow cytometry on day 42 after tumor inoculation. **a** The percentages of CD11c^+^ DC and CD11c^+^CD8^+^ DC subsets in the spleen of mice in each group. One typical flow cytometry result was shown for each group. **b**, **c** Statistical analysis of the percentages of DCs subpopulations. **d** The expression of CD80, CD86, or MHC-II on CD11c^+^ cells in the spleen of Ad vaccines-treated mice. **e**–**g** Statistical analysis of the percentages of CD80^+^CD11c^+^, CD86^+^CD11c^+^ or MHC-II^+^CD11c^+^ cells. Data were obtained from one representative experiment of three performed and presented as mean ± SD. The different significance was set at ***p < 0.001 and ****p < 0.0001. Multiple groups of comparison data were analyzed by one-way ANOVA.

Next, in order to further investigate the potential mechanism of the anti-tumor effect of the vaccine Ad-CAIX/Ad-PD-L1, we used flow cytometry to analyze the number and proportion of different immune cells infiltrated into the subcutaneous tumors of mice in each group. Compared with the Ad-CAIX group, the percentages and total numbers of tumor-infiltrating T cells, CD8^+^ T cells, and DCs were increased in the Ad-CAIX/Ad-PD-L1 combination group (Fig. 1e, f). However, there were no significant differences in the proportions and total numbers of CD4^+^ T cells, NK cells, and macrophages among the groups. These results indicated that combined immunization of Ad-CAIX and Ad-PD-L1 could effectively upregulate the infiltration of T cells, CD8^+^ T cells, and DCs in tumors, thereby exerting anti-tumor effects.

### Ad-CAIX/Ad-PD-L1 promoted the induction and maturation of DCs in vivo

DCs are the most important antigen-presenting cells (APCs) in the body, and they are responsible for the uptake, processing, and classification of antigens, and present antigen information to T cells, to initiate antigen-specific T cell immune responses. A previous study has demonstrated an increase in the number of CD11c^+^ cells and CD11c^+^CD8^+^ cells in the spleen after DNA vaccination^36^. To assess whether the vaccine Ad-CAIX/Ad-PD-L1 could enhance the induction and maturation of DCs in vivo, we investigated the proportions of DCs and CD8^+^ DCs in the spleen of mice in each group and detected the expression of co-stimulatory molecules (CD80, CD86) and antigen-presenting molecule (MHC-II). Compared with the Ad-CAIX group, the Ad-CAIX/Ad-PD-L1 combined group showed a significant increase in the percentages of CD11c^+^ cells and CD8^+^CD11c^+^ cells in spleens (Fig. 2a–c). Similarly, in the Ad-CAIX/Ad-PD-L1 combination treatment group, the proportions of CD80^+^CD11c^+^, CD86^+^CD11c^+^, and MHC-II^+^CD11c^+^ cells were remarkably augmented in the spleens (Fig. 2d–g). These results indicated that Ad-CAIX/Ad-PD-L1 could significantly increase the induction and maturation of DCs in vivo, and promote the uptake and presentation of tumor antigens, and thus activate the antigen-specific CD8^+^ T cell immune responses.

### Ad-CAIX/Ad-PD-L1 co-immunization enhanced antigen-specific CD8^+^ T cell immune responses in vivo

**Fig. 3:**
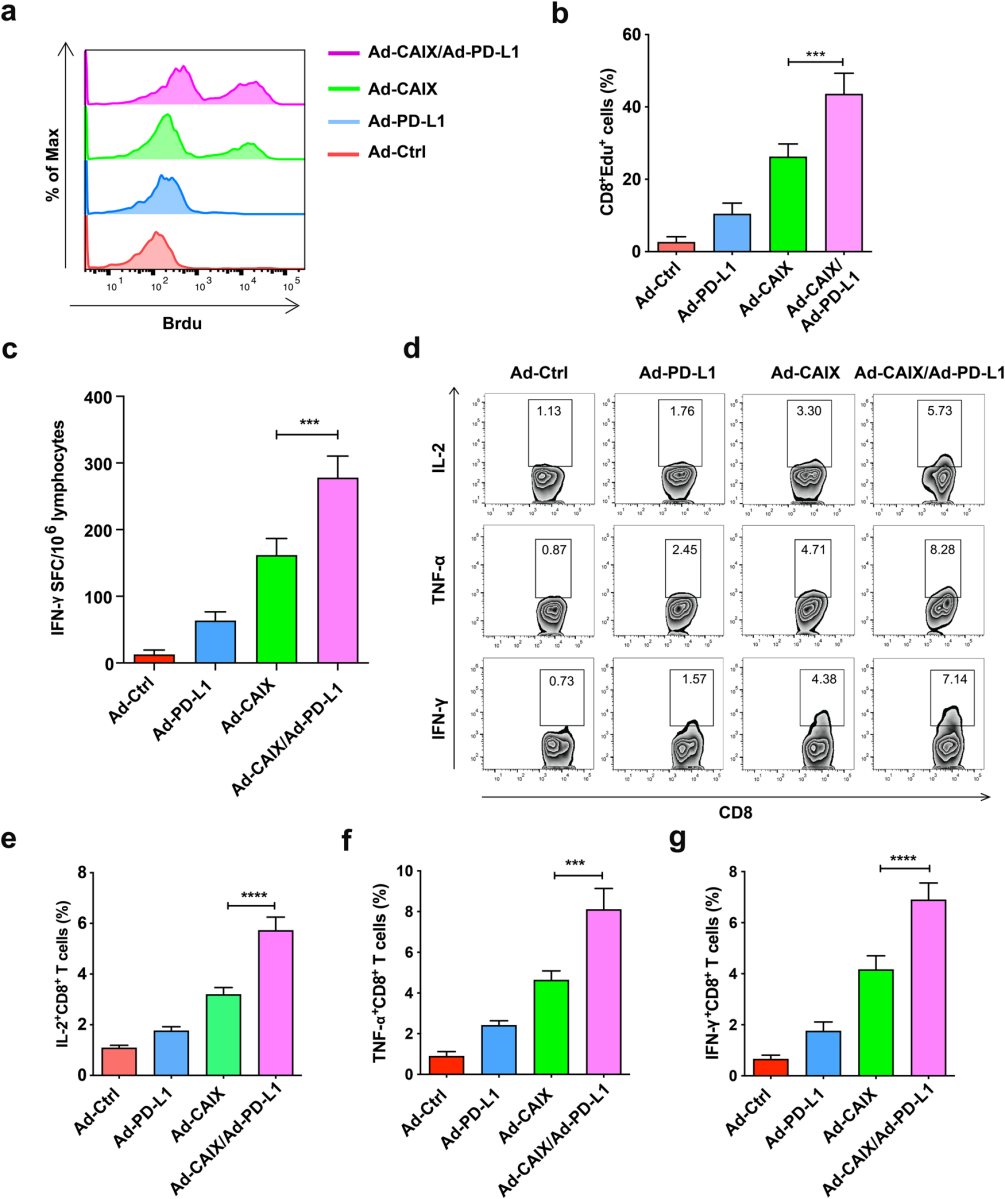
Stronger antigen-specific CD8^+^ T cell immune responses induced by the combined immunization of Ad-CAIX/Ad-PD-L1. **a**, **b** The proliferation of lymphocytes isolated from the spleens of mice in each group after continuous stimulation of CAIX protein (10 μg/ml) was detected by the EdU assay. **c** Detection of T lymphocytes secreting IFN-γ by ELISPOT assay. **d** After stimulation with CAIX protein, the proportions of IL-2^+^CD8^+^ T cells, IFN-γ^+^CD8^+^ T cells, and TNF-α^+^CD8^+^ T cells in splenocytes from each group were detected by flow cytometry. **e**–**g** Statistical analysis of the percentages of IL-2^+^CD8^+^ T cells, TNF-α^+^CD8^+^ T cells, and IFN-γ^+^CD8^+^ T cells in **d**. Data presented as mean ± SD from one representative experiment of three performed. The different significance was set at ***p < 0.001 and ****p < 0.0001. Multiple groups of comparison data were analyzed by one-way ANOVA.

CD8^+^ cytotoxic T lymphocytes are the main effector cells in vivo that exert specific cytotoxic effects and play a critical role in anti-tumor effects. In order to evaluate whether Ad-CAIX/Ad-PD-L1 can enhance the CD8^+^ T cell-mediated immune responses, we isolated the lymphocytes from spleens of mice in each group and examined the proliferation capacity and Th1 cytokines produced by CD8^+^ T cells after stimulation with CAIX protein in vitro. Compared to the Ad-CAIX treatment group, CD8^+^ T cells in the Ad-CAIX/Ad-PD-L1 combined immunization group showed a stronger proliferation capacity (Fig. 3a, b). The ELISPOT assay was performed to detect the IFN-γ level secreted by CD8^+^ T cells, and a similar increasing trend was observed in the Ad-CAIX/Ad-PD-L1 co-immunization group (Fig. 3c). Additionally, we performed intracellular staining of CD8^+^ T cells, and the result showed that the proportions of IL-2^+^CD8^+^ T cells, IFN-γ^+^CD8^+^ T cells, and TNF-α^+^CD8^+^ T cells in the Ad-CAIX/Ad-PD-L1 combination group were significantly higher than those in the Ad-CAIX treatment group (Fig. 3d–g). The above experimental results demonstrated that Ad-CAIX/Ad-PD-L1 vaccine significantly enhanced the antigen-specific CD8^+^ T cell immune responses in vivo.

### The anti-tumor effects triggered by Ad-CAIX/Ad-PD-L1 mainly depended on the immune responses mediated by multifunctional CD8^+^ T cells

**Fig. 4:**
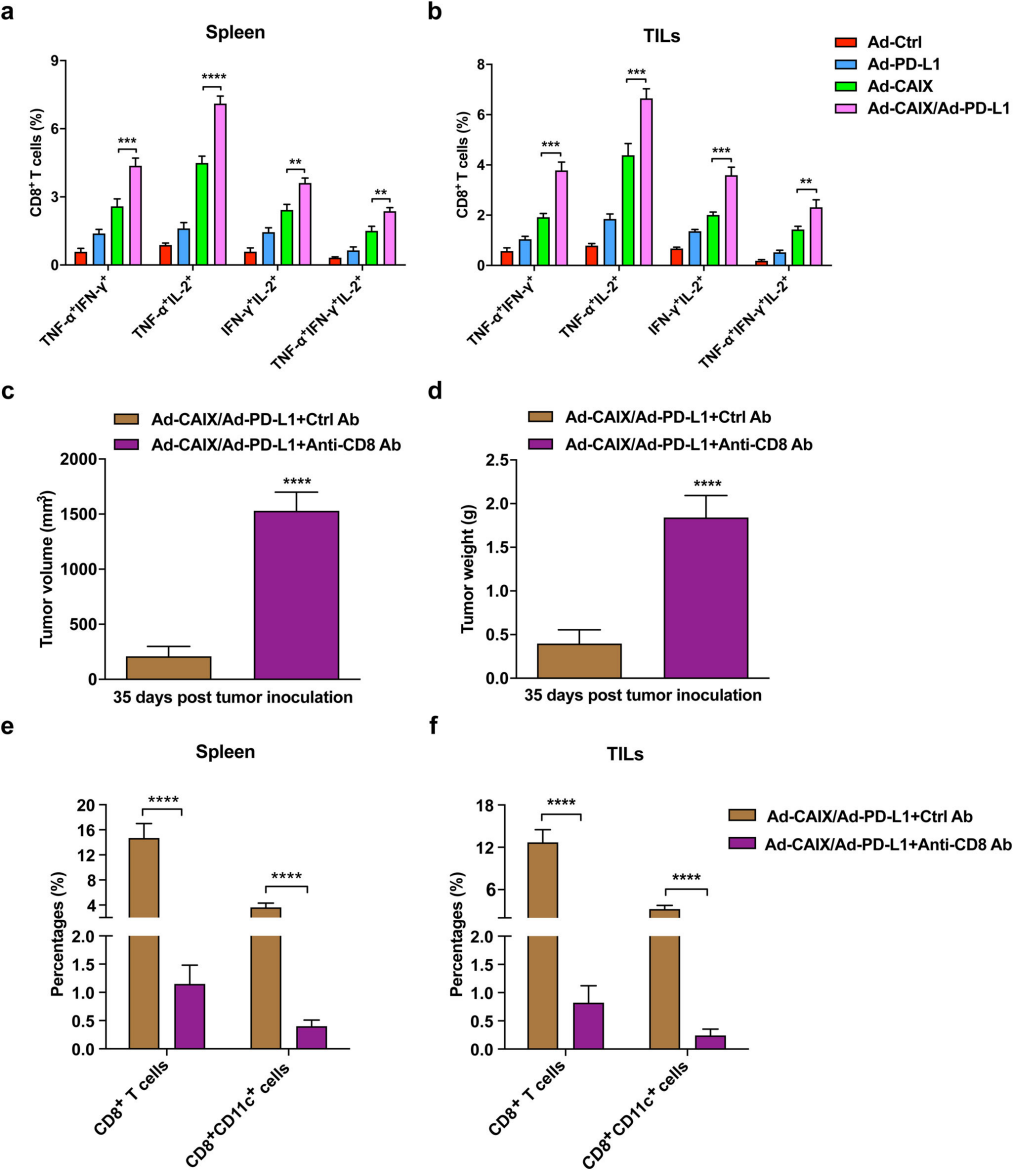
Ad-CAIX/Ad-PD-L1 enhanced the induction of multifunctional CD8^+^ T cells and relied on CD8^+^ T cells to exert anti-tumor effects. **a**, **b** The proportions of T cell subsets of TNF-α^+^IFN-γ^+^CD8^+^, TNF-α^+^IL-2^+^CD8^+^, IFN-γ^+^IL-2^+^CD8^+^, and TNF-α^+^IFN-γ^+^IL-2^+^CD8^+^ in the splenocytes and TILs in each group. **c**, **d** The tumor volumes and weights of mice in each group at the endpoint of the CD8^+^ T cell depletion assay. **e**, **f** The percentages of CD8^+^ T cells and CD8^+^ DCs in spleens and TILs in each group in the CD8^+^ T cell depletion assay. Data were from one representative experiment of three performed and presented as mean ± SD. The different significance was set at **p < 0.01, ***p < 0.001, and ****p < 0.0001. Multiple groups of comparison data were analyzed by one-way ANOVA or two-tailed independent Student’s t-test was performed for two groups of comparison data, respectively.

Multifunctional CD8^+^ T cells, as the critical effector cells of protective immunity, can secrete various cytokines (IFN-γ, TNF-α, IL-2, etc.), playing an important role in the anti-tumor effect. Therefore, we further tested the proportion of CD8^+^ T cells that secreted two or more cytokines in the spleen lymphocytes and the tumor-infiltrating T cells of the immunized mice in each group. The percentages of TNF-α^+^IFN-γ^+^CD8^+^, TNF-α^+^IL-2^+^CD8^+^, IFN-γ^+^IL-2^+^CD8^+^, and TNF-α^+^IFN-γ^+^IL-2^+^CD8^+^ T cells were significantly increased in the splenic lymphocytes of the Ad-CAIX/Ad-PD-L1 co-immunotherapy group compared with those of the Ad-CAIX treatment group (Fig. 4a). Similar results were observed with the detection of tumor-infiltrating T cells in subcutaneous tumors (Fig. 4b). These data indicated that the induction of antigen-specific multifunctional CD8^+^ T cells was significantly enhanced by the combination of Ad-CAIX and Ad-PD-L1. Furthermore, the CD8^+^ T cell depletion assay, using anti-CD8 mAb to deplete CD8^+^ T cells in vivo, was performed to determine whether functional CD8^+^ T cells were essential for the anti-tumor activity induced by the Ad-CAIX/Ad-PD-L1 vaccine. After CD8^+^ T-cell depletion, the subcutaneous tumors significantly increased in volume and weight compared to controls (Fig. 4c, d), and the vaccine-induced anti-tumor effects disappeared. Flow cytometry analysis showed that the ratios of CD8^+^ T cells and CD8^+^ DCs in the CD8-depleted group were significantly down-regulated in the splenocytes and tumor (Fig. 4e, f). These results indicated that Ad-CAIX/Ad-PD-L1 could enhance the antigen-specific multifunctional CD8^+^ T cell response, which was critical for anti-tumor effects induced by the vaccine.

### Ad-CAIX/Ad-PD-L1 prevented tumor lung metastasis of renal carcinoma by promoting tumor-specific CD8^+^ T cell immune responses

**Fig. 5:**
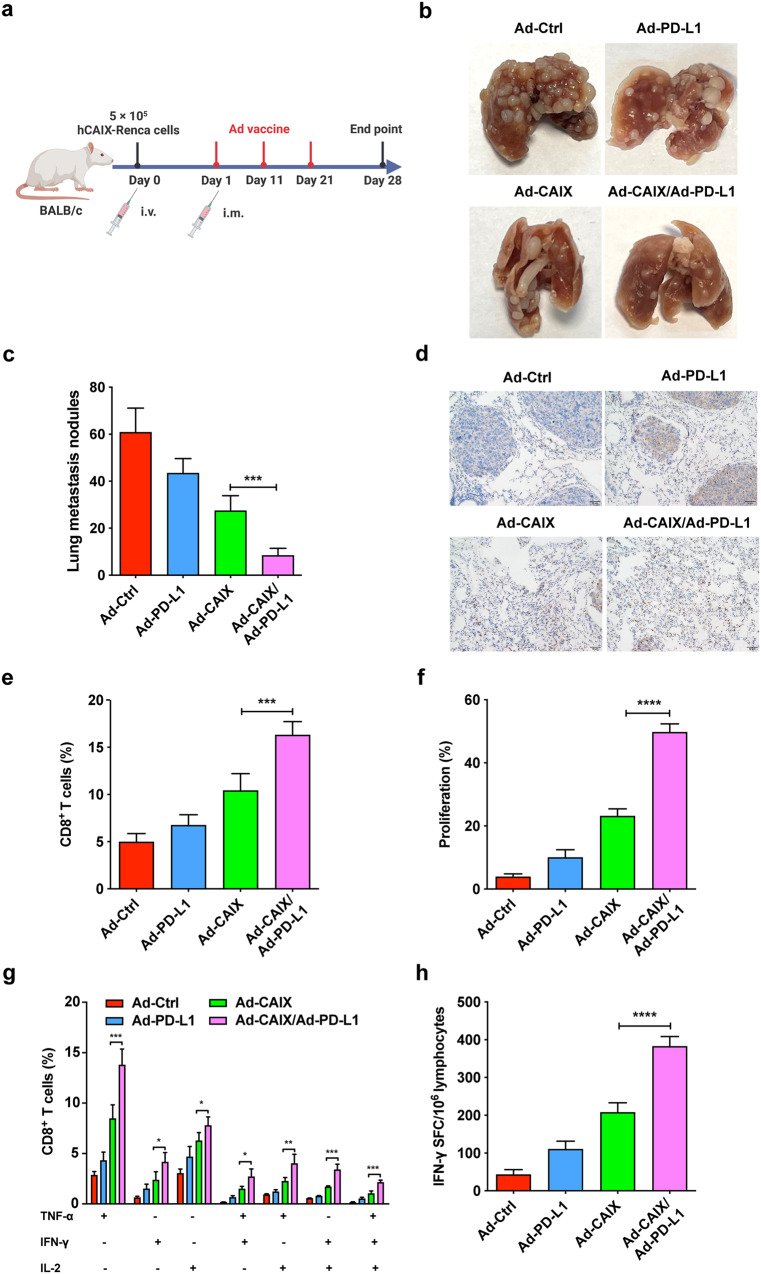
The therapeutic effects of Ad-CAIX/Ad-PD-L1 in the hCAIX-Renca lung metastasis model. **a** Schematic diagram illustrating the establishment of lung metastasis model and the experimental design. **b** Representative images of metastatic nodules on lung surface of mice each group. **c** Quantitative statistical analysis of the number of nodules on the lung surface. **d** Immunohistochemistry was used to detect CD8^+^ T cell infiltration in the lungs of mice in each group (scale bar, 50 μm). **e** Percentages of CD8^+^ T cells in infiltrating immune cells from the lung tumor tissues of mice in each group. **f** The proliferation of CD8^+^ T cells was detected by the EdU assay. **g** The percentages of CD8^+^ T cells expressing TNF-α, IFN-γ, or IL-2 in stimulated splenocytes. **h** IFN-γ- secreting T lymphocyte cells were detected by ELISPOT assay. Data were from one representative experiment of three performed and presented as mean ± SD. The different significance was set at **p < 0.01 and ***p < 0.001. Multiple groups of comparison data were analyzed by one-way ANOVA.

A lung metastasis model was established to evaluate the therapeutic effect of the Ad-CAIX/Ad-PD-L1 vaccine on lung metastasis of renal carcinoma. A total of 5 × 10^5^ hCAIX-Renca cells were injected through the tail vein, and mice were randomly divided into four groups (Ad-Ctrl, Ad-PD-L1, Ad-CAIX, and Ad-CAIX/Ad-PD-L1) and immunized with Ad vaccines by intramuscular injection (Fig. 5a). After the mice were sacrificed, the lungs were removed, and the number of nodules on the lung surface in each group was counted. The number of metastatic nodules on the lung surface was significantly reduced in the Ad-CAIX/Ad-PD-L1 combined group compared to the Ad-CAIX treatment group, (Fig. 5b, c). Immunohistochemistry and flow cytometry results of lung tissues showed that the infiltration of CD8^+^ T cells in the Ad-CAIX/Ad-PD-L1 combined group was significantly higher than that in other groups (Fig. 5d, e). Similar to the results observed in the subcutaneous tumor model, CD8^+^ T cells in the Ad-CAIX/Ad-PD-L1 combined group also exhibited stronger proliferation ability compared to the Ad-CAIX group (Fig. 5f). Additionally, multifunctional T cell assay showed that the proportions of TNF-α^+^IFN-γ^+^CD8^+^, TNF-α^+^IL-2^+^CD8^+^, IFN-γ^+^IL-2^+^CD8^+^, and TNF-α^+^IFN-γ^+^IL-2^+^CD8^+^ T cells in the Ad-CAIX/Ad-PD-L1 combined group were significantly increased in splenocytes cultured in vitro (Fig. 5g). Similarly, a significant increase in T cells secreting IFN-γ was also observed in the Ad-CAIX/Ad-PD-L1 combination group (Fig. 5h). These results indicated that Ad-CAIX/Ad-PD-L1 inhibited the lung metastasis of the tumor by enhancing the anti-tumor immune responses mediated by CD8^+^ T cells in the hCAIX-Renca lung metastasis model.

### Ad-CAIX/Ad-PD-L1 suppressed the tumor progression in the hCAIX-Renca renal orthotopic model

**Fig. 6:**
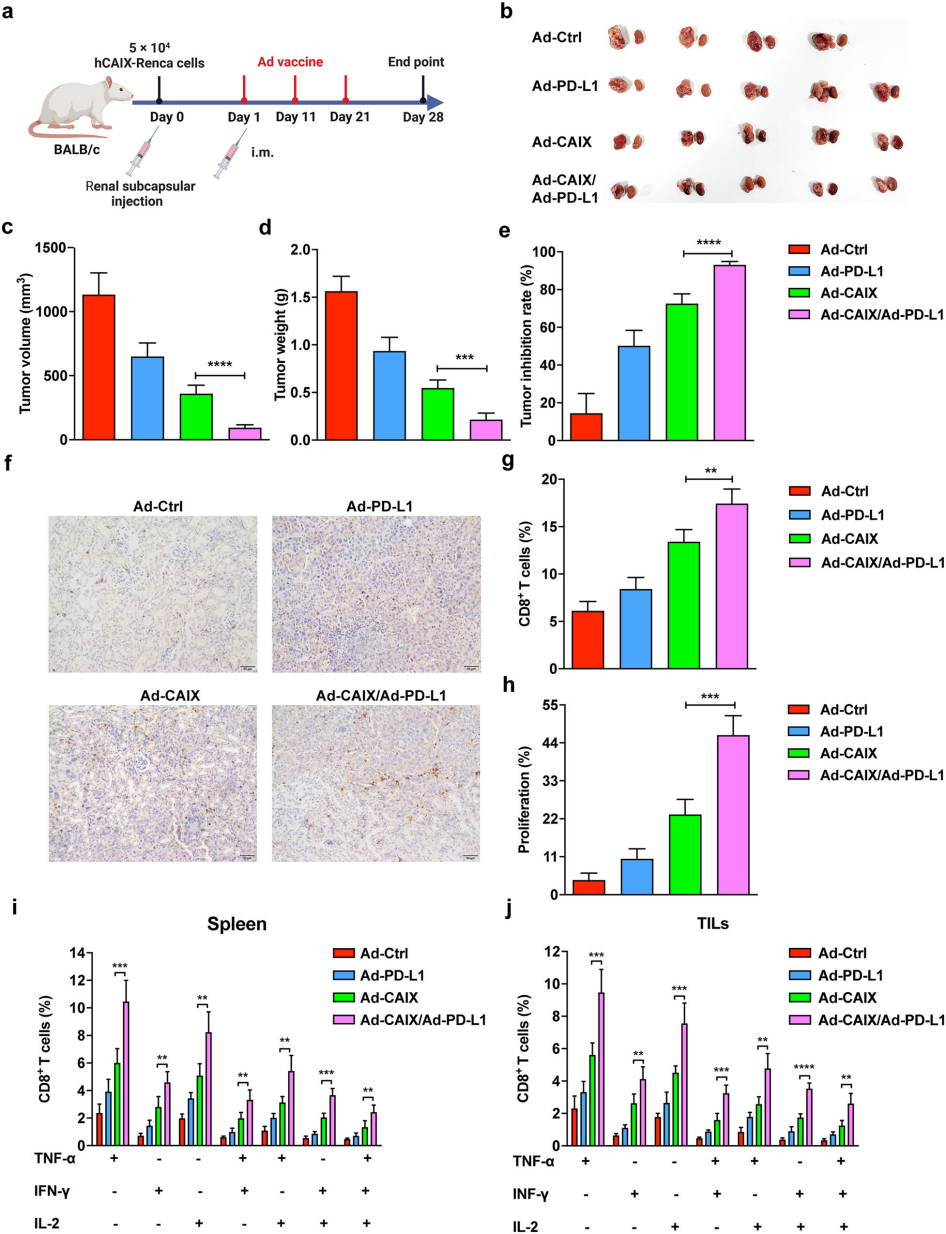
Therapeutic effects of Ad-CAIX/Ad-PD-L1 in the hCAIX-Renca renal orthotopic model. **a** Schematic diagram of the establishment of hCAIX-Renca renal orthotopic model and vaccine immunization. **b** Representative images of bilateral kidneys of mice in each group at the end of the experiment (unilateral tumor). **c** Tumor volumes. **d** Tumor weights. **e** Statistical analysis of tumor inhibition rate. **f** Infiltration of CD8^+^ T cells detected by IHC in renal tumor tissues (scale bar, 50 μm). **g** Determination of the proportion of infiltrating CD8^+^ T cells in renal tumors by flow cytometry. **h** The proliferation of CD8^+^ T cells was detected by the EdU assay. **i**, **j** Proportions of TNF-α^+^CD8^+^, IL-2^+^CD8^+^, IFN-γ^+^CD8^+^, TNF-α^+^IFN-γ^+^CD8^+^, TNF-α^+^IL-2^+^CD8^+^, IFN-γ^+^IL-2^+^CD8^+^ and TNF-α^+^IFN-γ^+^IL-2^+^CD8^+^ T cell subsets of mice in spleens and TILs from each group. Individual experiments were performed three times, and results from one representative experiment were shown for each group of mice. Data are means ± SD. **p < 0.01, ***p < 0.001, and ****p < 0.0001. Multiple groups of comparison data were analyzed by one-way ANOVA.

**Fig. 7:**
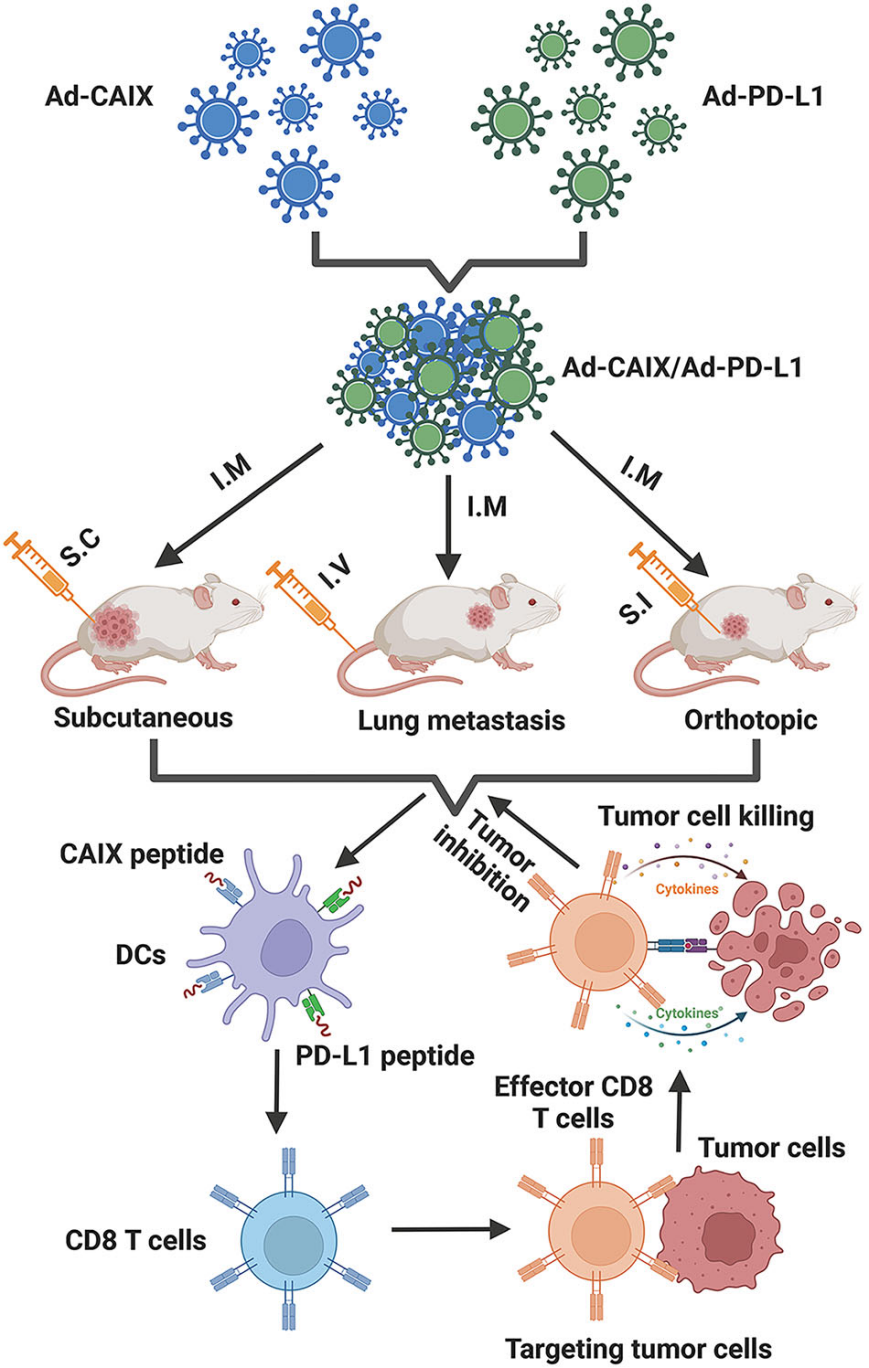
The schematic illustration of the therapeutic effect of Ad-CAIX/Ad-PD-L1 in renal tumor models. Ad-CAIX and Ad- PD-L1 vaccines were prepared and expressed in vitro. Three tumor models, including the subcutaneous, lung metastasis, and orthotropic tumor, were established, and intramuscular Ad vaccine immunization was performed. Ad-CAIX/Ad-PD-L1 could effectively enhance the induction and maturation of DCs and DC subsets, and promote strong tumor-specific CD8^+^ T cell immune responses. Ad-CAIX/Ad-PD-L1 vaccine could significantly inhibit tumor growth or lung metastasis in three models via DCs-mediated CD8^+^ T cell anti-tumor responses.

The therapeutic effect of the Ad-CAIX/Ad-PD-L1 vaccine was further evaluated in primary renal tumors of established renal orthotopic models in BALB/c mice. A total of 5 × 10^4^ hCAIX-Renca cells were inoculated under the unilateral renal capsule of mice, and the mice were randomly grouped and immunized on day 1 post-tumor inoculation. The mice in each group were immunized by intramuscular injection once every 10 days and were immunized three times in total (Fig. 6a). Renal tumors in the Ad-CAIX/Ad-PD-L1 combination group were significantly smaller in terms of volumes and weights compared to the Ad-CAIX group (Fig. 6b–d), and the tumor inhibition rate was remarkably increased compared with other groups (Fig. 6e). Renal tumor immunohistochemistry and flow cytometry results showed that Ad-CAIX/Ad-PD-L1 combination group had a higher CD8^+^ T-cell infiltration than the other groups (Fig. 6f, g). Similar to the previous results, the proliferation of Edu^+^CD8^+^ T cells was significantly increased in the Ad-CAIX/Ad-PD-L1 combination group (Fig. 6h). Furthermore, compared with the Ad-CAIX group, the test results of multifunctional T cells showed that the proportions of TNF-α^+^IFN-γ^+^CD8^+^, TNF-α^+^IL-2^+^CD8^+^, IFN-γ^+^IL-2^+^CD8^+^, and TNF-α^+^IFN-γ^+^IL-2^+^CD8^+^ T cells were strikingly elevated in the activated lymphocytes from spleens of Ad-CAIX/Ad-PD-L1 combination group (Fig. 6i). Flow cytometry of infiltrating lymphocytes within the tumor also revealed similar findings (Fig. 6j). Collectively, Ad-CAIX/Ad-PD-L1 vaccine could effectively inhibit tumor growth or lung metastasis in the subcutaneous, lung metastasis, and orthotropic tumor models by DCs-mediated CD8^+^ T cell anti-tumor immune responses (Fig. 7).

## Discussion

Tumor vaccines have the potential to induce a specific and long-lasting immune response against tumor antigens, making them a promising therapeutic strategy for cancer immunotherapy^7^. In recent years, virus-based gene therapy, with Ad vectors being the most commonly used gene therapy vectors, has emerged as an effective cancer immunotherapy^38^. Replication-deficient viral vectors share some of the characteristics of live attenuated vaccines that deliver antigen genes into cells, using potent exogenous promoters to promote antigen expression in vivo^12^. In this study, we designed an Ad-mediated tumor DNA vaccine that contained two replication-deficient Ads: Ad-CAIX and Ad-PD-L1, which encoded the CAIX and PD-L1 respectively. We evaluated the anti-tumor effects of Ad-CAIX/Ad-PD-L1 vaccine in subcutaneous, orthotopic, and metastatic renal cancer models. However, the use of human-CAIX in our studies is a limitation in the current model. To further verify the therapeutic effects of the vaccine, we plan to address these rapidly growing tumors by studying the timing of vaccine administration (e.g. administration on day 1 after tumor challenge). This approach may help overcome the limitation and improve the efficacy of the vaccine, particularly in the context of rapidly growing tumors.

Given the low immunogenicity of CAIX antigen, we employed Ad as a vector and leveraged the "self-adjuvant" properties of Ads to enhance the immunogenicity of CAIX. Simultaneously, we designed another replication-deficient Ad carrying the immune checkpoint PD-L1 to induce a specific immune response against PD-L1 in vivo. The Ad-CAIX was combined with Ad-PD-L1 to enhance the anti-tumor effect. Our Ad-CAIX/Ad-PD-L1 vaccine efficiently expressed the entire CAIX or PD-L1 protein as antigens. While this provides an advantage to express a protein that is structurally like that expressed by tumor cells and cover all possible antigen epitopes. It will be important as we move forward to identify key antigenic epitopes involved in our therapeutic response to understand the immune response generated by our vaccine and to better elucidate the mechanism of action of Ad-CAIX/Ad-PD-L1. The vaccine of Ad-CAIX/Ad-PD-L1 showed significant inhibitory effects on tumor growth in three tumor models. Ad-CAIX/Ad- PD-L1 could stimulate the induction and maturation of DCs in vivo and further enhance the CD8^+^ T cell-mediated specific anti-tumor immune response. Moreover, we observed that the anti-tumor effects of Ad-CAIX/Ad-PD-L1 were primarily mediated by inducing multifunctional CD8^+^ T cells in vivo to elicit the immune responses. This vaccine offers a new approach and strategy for immunotherapy for renal cancer and provides an experimental foundation for its clinical application.

PD-L1 is a cell surface glycoprotein and one of the co-inhibitory members of the B7 family^33^. It is over-expressed in RCC and is associated with tumor invasion and poor prognosis. PD-L1, when combined with PD-1, can inhibit the proliferation and differentiation of T cells and negatively regulate the function of T cells, which is one of the important mechanisms of tumor immune escape^39^. Multiple experimental studies have shown that PD-L1-based protein or DNA vaccines can inhibit tumor growth in mouse models of various cancers, including myeloma^35,36^, colon cancer^37^, breast cancer^37^, or melanoma^40^. Not only do tumor cells express PD-L1 highly, but some regulatory immune cells in the tumor microenvironment also express PD-L1, such as tumor-associated DCs (TADCs) and myeloid-derived suppressor cells (MDSCs). PD-L1-specific T cells are able to recognize and eliminate both cancer cells and regulatory immune cells. Therefore, activating PD-L1-specific T cells by vaccines can directly target immunosuppressive pathways in the tumor microenvironment, modulate the immune microenvironment and potentially alter the tolerance of the immune system to tumor antigens^41^. Our experiment showed that the combination of Ad-CAIX and Ad-PD-L1 was more effective in the three animal models than Ad-CAIX monotherapy group. The experimental results showed that the combined immunity of Ad-PD-L1 and Ad-CAIX could enhance the anti-tumor effect. The Ad vaccine has a high safety profile since the Ad genome is not integrated into the host cell genome, and the Ads used in our experiments are replication-deficient. In the short term, no obvious immunotoxicity and inflammatory damage were observed in mice immunized with various vaccines. However, the safety of Ad-PD-L1/Ad-CAIX was not evaluated in the long term. Ad-vaccine-mediated PD-L1 may lead to long-term adaptive immunity, which ultimately has severe autoimmune consequences. Therefore, further study is need to evaluate the safety of this vaccine through long-term observation.

DCs, as professional antigen-presenting cells, play an important role in the innate and adaptive immune response. Immature DC captures antigens at the inflammatory site, matures and migrates to the draining lymph nodes, further processes antigens into peptides, and cross-presents captured antigens on MHC-I molecules to activate the CD8^+^ T cell responses^42^. CD11c^+^ DCs mainly induces specific CD8^+^ T cell responses, Th1 reactions, and enhanced CTL activity^43^. Therefore, enhanced induction of CD11c^+^ DCs can improve the anti-tumor effect of DNA vaccines. In our study, the combined immunity of Ad-CAIX/Ad-PD-L1 significantly upregulated the proportion of CD11c^+^ DCs subpopulations and simultaneously increased the expression of DC surface activation markers CD80, CD86, and MHC-II. Our results showed that the Ad-CAIX/Ad-PD-L1 vaccine could enhance the induction of CD11c^+^ DCs and promote their maturation in vivo.

The purpose of the vaccine is to activate the immune system to remove tumor cells, which is closely related to the induction of antigen-specific CD8^+^ T cell immune responses. In this experimental study, we observed that the Ad-CAIX/Ad-PD-L1 vaccine could effectively enhance the CD8^+^ T cell immune response in vivo. Furthermore, we used mAb to delete CD8^+^ T cells in vivo. The experimental results showed that in the CD8^+^ T cell deletion group, the Ad-CAIX/Ad-PD-L1 no longer inhibited tumor growth in mice, indicating that CD8^+^ T cells played a key role in anti-tumor immunity. The above results indicated that the anti-tumor effect of the Ad-CAIX/Ad-PD-L1 vaccine mainly depended on the immune response mediated by CD8^+^ T cells. Additionally, Ad vaccines can indirectly activate humoral immunity. A previous study showed that the preventive PD-L1 protein vaccine was able to induce high titers of antibodies against PD-L1 and efficiently prevented tumor growth^35^. The vaccine-induced PD-L1 antibody might play an essential role in helping and stimulating tumor-specific CD8^+^ T cells. The PD-L1 specific antibody can also block both PD-L1 positive tumor cells as well as PD-L1 positive immunosuppressive cells, further activating CD8^+^ T cell immune responses. Therefore, we will further investigate the effect of vaccine-induced antibodies on the immune response of CD8^+^ T cells in future studies.

It is well known in the field that the antigen specific cellular responses generated by Ad5 are not well boosted preclinically and clinically after repeated administrations. We are considering several strategies to address this limitation to our current therapeutic design, such that will limit the need for repeated immunizations by combining with other technology for heterologous prime-boost for future clinical applications. We also understand that these and other approaches will need active research in areas of setting our optimal frequency and timing of vaccine administration to maximize the efficacy of our approach. Furthermore, modifications to the vector backbone, such as deletion of certain viral genes, can reduce the immune response to the vector and improve its safety profile. These advancements in adenovector technology have shown promising results in preclinical and clinical studies and may be a viable alternative for the development of therapeutic vaccines in the future. Additionally, we also plan to shift Ad5 based vaccines to the next generation of adenovector technology in our clinical studies. We understand that clinical translation is a lengthy process, but we are working hard towards this goal.

In summary, our experimental results showed that the Ad-CAIX/Ad-PD-L1 vaccine can effectively inhibit the growth of subcutaneous tumors, lung metastases, and orthotopic tumors of renal carcinoma in mice. The vaccine promoted the induction and maturation of DCs in vivo and further promoted the multifunctional CD8^+^ T cell-mediated CTL immune response. Therefore, the Ad-CAIX/Ad-PD-L1 vaccine may provide a new strategy for the non-surgical treatment of kidney cancer.

## Materials and methods

### Animals

Six weeks old female BALB/c mice were purchased from the Laboratory Animal Center of Xuzhou Medical University (Xuzhou, China) and housed under specific-pathogen-free (SPF) conditions with standard temperature and humidity. All the animal procedures and protocols were authorized by the Laboratory Animal Ethical Committee of Xuzhou Medical University. All performances accord with the guidelines for the Care and Use of Laboratory Animals of Xuzhou Medical University.

### Cell lines and cell culture

Human embryonic kidney (HEK) 293 cell line was obtained from ATCC and cultured in DMEM medium (Gibco, Invitrogen) supplemented with 10% FBS (ExCell Bio), 100 μg/ml streptomycin (Sangon Biotech), and 100 U/ml penicillin (Sangon Biotech) at 37 °C in a humidified incubator containing 5% CO_2_. The mouse renal carcinoma cell line (Renca) was acquired from Cobioer Biosciences (Nanjing, China) and verified by an analysis certificate. The stable cell line hCAIX-Renca was established and stored in our laboratory^29^. Renca or hCAIX-Renca was cultured in RPMI-1640 medium (Gibco, Invitrogen) containing 10% FBS, 100 U/ml penicillin, 100 μg/ml streptomycin, 1 mM sodium pyruvate solution (Sigma), 1× MEM non-essential amino acid solution (Sigma), and 2mM L-glutamine (Sigma).

### Construction and expression of Ad vaccines

In this study, the used two adenovirus shuttle vectors, pCA13 and E1A-deleted Ad5 backbone vector pPE3, were kindly donated by Professor Lin Fang of Xuzhou Medical University. The gene fragments of hCAIX were amplified from pcDNA3.1-hCAIX previously constructed and stored in our laboratory by PCR using primers for hCAIX (Forward primer, 5′-TTAAGCTTATGGCTCCCCTGTGCCCCAGC-3′; Reverse primer, 5′-TGCTCTAGAGGCTCCAGTCTCGGCTACCTCTGCTG-3′). The full-length fragments of mouse PD-L1 were cloned from pEnCMV-mPD-L1 (Miaoling Bio) by using primers for mPD-L1 (Forward primer, 5′-TTAAGCTTATGAGGATATTTGCTGGCATTATATTCACAG-3′; Reverse primer, 5′-TGCTCTAGACGTCTCCTCGAATTGTGTATCATTTCG-3′). The PCR sample was subcloned into the Hind III and Xba I sites of the pCA13 vector. Then, the plasmid pCA13-hCAIX or pCA13-mPD-L1 was sequenced to ensure its successful construction, and it was co-transfected into HEK293 cells by homologous recombination with pPE3 to produce Ad-CAIX or Ad-PD-L1 (Supplementary Fig. 1a). About 10–14 days after transfection, the plaque was observed (Supplementary Fig. 1b). Ads were purified on a large scale by density gradient ultracentrifugation with cesium chloride. And titer was determined by TCID50 assays. The control Ad (Ad-Ctrl) constructed by the above method using pCA13 and pPE3 was also developed in HEK293 cells.

HEK293 cells were infected with Ad-CAIX or Ad-PD-L1, and viral genomic DNA was extracted from the infected supernatant by QIAGEN genomic DNA kit. Thereafter, PCR was carried out with the amplification primers of hCAIX or mPD-L1. The purity of the DNA was verified by electrophoresis at 100 V for 45 min on 2% agarose gel. Subsequently, the gel was analyzed at the wavelength of 312 nm by using the FireReader gel imaging system (FireReader) (Supplementary Fig. 1c, d). In addition, flow cytometry was performed to verify the validity of Ad-PD-L1 (Supplementary Fig. 1e, f) or Ad-CAIX (Supplementary Fig. 1g, h).

### Animal models and vaccination

To establish the subcutaneous tumor models, 2 × 10^5^ hCAIX-Renca cells suspended in 100 μl of PBS were subcutaneously implanted into the right flank of mice on day 0 and randomly divided into four groups (*n* = 5). On the seventh day post-tumor inoculation, mice were intramuscularly immunized with Ad-Ctrl, Ad-PD-L1, or Ad-CAIX, and Ad-CAIX/Ad-PD-L1 on the same side of the tumors, respectively. The dose of 3 × 10^8^ PFU was used for each Ad vaccine. To ensure the same amount of Ad vaccine, animals immunized with Ad-Ctrl, Ad-PD-L1 or Ad-CAIX were received with another Ad-Ctrl vaccine. After the first immunization, the mice were boost immunized on day 10 and day 20. Tumors were measured with a digital caliper once a week. The following formula was used to evaluate the tumor volume: V (mm^3^) = (length × width^2^)/2. On day 42 after tumor inoculation, mice were sacrificed and tumor tissues were surgically excised and weighed. For the lung metastasis model, 5 × 10^5^ hCAIX-Renca cells were injected into the tail vein on day 0. The mice were given an intramuscular injection of the same amount of vaccine on day 1 and repeated immunizations on day 11 and 21 post-tumor inoculation. On day 28 after tumor inoculation, mice were sacrificed, and the lungs were surgically excised. Metastatic nodules in the lung tissue were quantified. In order to establish orthotopic tumor models, the mice were anesthetized with 5% chloral hydrate, and a subcapsular injection (S.I) was performed in the unilaterally exposed kidney, in which 5 × 10^4^ hCAIX-Renca cells were suspended in 10 μl of PBS. Then, each layer of the surgical incision was sutured. Mice were intramuscularly immunized with the treatment consistent with the lung metastasis models. At the end of the study, tumor volume and weight were evaluated.

### Preparation of single-cell suspension

The spleens or tumor tissues were harvested and gently ground in PBS buffer by using a 5 ml clean syringe plunger. The released cells were gathered, resuspended, and then filtered through 70 μm nylon mesh to removed debris. After centrifugation at 400 x *g* for 5 min, red blood cells were lysed with red blood cell lysis buffer. To prepare a single-cell suspension, the remaining cells were collected and resuspended in an RPMI-1640 culture medium. Tumor-infiltrating leukocytes (TILs) were separated using a 33% Percoll (VicMed) gradient.

### Flow cytometry analysis

For cell surface staining, cells were incubated with the following antibodies: PE-conjugated anti-human CAIX (R&D Systems, Cat. FAB2188P, 1:100), PE-conjugated anti-mouse CD3ε (BioLegend, Cat. 100308, 1:100), APC-conjugated anti-mouse PD-L1 (BioLegend, Cat. 124312, 1:100), APC-conjugated anti-mouse CD11c (BioLegend, Cat. 117310, 1:100), PerCP-Cy5.5-conjugated anti-mouse CD4 (BioLegend, Cat. 116012, 1:100), PerCP-Cy5.5-conjugated anti-mouse CD8α (BioLegend, Cat. 100734, 1:100), FITC anti-mouse CD49b (BioLegend, Cat. 108906, 1:100), PerCP-conjugated anti-mouse F4/80 (BioLegend, Cat. 123126, 1:100), FITC-conjugated anti-mouse CD11b (BioLegend, Cat. 101206, 1:100) for 1 h at 4 °C. The total isolated cells were counted using a cell counter, and then some cells were stained and detected by flow cytometry to obtain the percentage of immune subsets. The total number of cells was multiplied by the percentage of each cell subpopulation to arrive at their cell count.

For intracellular cytokine staining, splenocytes were resuspended in a 12-well plate at a density of 4 × 10^6^/ml and stimulated with 10 μg/ml recombinant human CAIX protein for 72 h. Cells were also incubated with 500 ng/ml Ionomycin (Sigma-Aldrich), 50 ng/ml PMA (Sigma-Aldrich), and 5 μg/ml Brefeldin A (BFA, eBioscience) at 37 °C and 5% CO_2_ for the last 5 h. Cells were collected and washed twice with PBS. Cell surface staining was performed using anti-mouse PerCP-Cy5.5-conjugated anti-CD8α, followed by intracellular staining with APC-conjugated anti-mouse IFN-γ (Biolegend, Cat. 505810, 1:100), FITC-conjugated anti-mouse TNF-α (Biolegend, Cat. 506304, 1:100), and PE-conjugated anti-mouse IL-2 (Biolegend, Cat. 503808, 1:100). All data were obtained on a BD FACSCanto II (BD Biosciences) or Cytek® Northern Lights (Cytek Biosciences) and analyzed using FlowJo software (Tree Star Inc.).

### Detection of CD8 T-cell proliferation

Lymphocytes were resuspended in a culture medium supplied with IL-2 (50 U/ml) and recombinant CAIX protein (10 μg/ml), seeded at a density of 1 × 10^6^ cells/well onto 48-well flat-bottom tissue culture plates, and incubated in a humidified incubator with 5% CO_2_ at 37 °C. After 72 h, the culture medium was refreshed, and then the culture was continued for 48 h. The proliferation analysis was conducted using BeyoClick™ EdU Cell Proliferation Kit with Alexa Fluor 647 (Beyotime). In brief, lymphocytes were incubated with 10 μM EdU at 37 °C for 2 h, collected, and stained with PerCP-Cy5.5-conjugated anti-CD8α for 30 min. The cells were then fixed, permeabilized, and rinsed before being exposed to 100 μl of the click reaction cocktail for 30 min. Finally, the cells were washed with the permeabilization buffer three times. Flow cytometry was used to analyze the percentages of EdU^+^ cells in CD8^+^ T cells, and the proliferation rate was calculated.

### ELISPOT assay

The mouse IFN-γ T cell enzyme-linked immune-spot (ELISPOT) monochromatic enzyme kit (eBioscience) was used to perform this assay. First, the ELISPOT plate was coated with an anti-IFN-γ antibody and left at 4 °C overnight. Then, it was blocked with RMPI-1640 containing 10% FBS for 2 h. Lymphocytes (1 × 10^6^ cells/well) were suspended in 200 μl RMPI-1640 medium and seeded into the plate. The cells were then stimulated with recombinant CAIX protein (10 μg/ml) and IL-2 (50 U/ml) and incubated in a 5% CO_2_ incubator at 37 °C for 72 h. The color was developed after sequential incubation with a biotinylated detection antibody, streptavidin-HRP, and AP-colorimetric substrate (eBioscience). The spot-forming cells (SFC) were enumerated using an immunospot analyzer (AID).

### CD8 T cell depletion

To deplete CD8^+^ T cells, mice were injected intraperitoneally (i.p) with 0.5 mg/mouse anti-mouse CD8α mAb (BioXCell, Cat. BE0004-1) on day 2 before the first dose of vaccine was administered, and repeated antibody injections on day 5 and day 12. The efficacy of cell depletion was confirmed by flow cytometry analysis.

### IHC staining

Tissues were fixed with formalin, embedded in paraffin, and sectioned. The sections were heated to 95 °C in 10 mmol/L sodium citrate (pH 6.0) for 30 min and then treated with 3% hydrogen peroxide for 1 h. The deparaffinized tissue slides retrieved epitopes of the rehydrated sections. The slides were blocked with 10% Bovine Serum Albumin (BSA) for 2 h and then incubated with rat anti-mouse CD8 antibody (eBioscience, Cat. 14-0081-82, diluted at 1:100) and goat anti-rat antibody (Zhongshan Biotech, Cat. PV9004). Hematoxylin was used for the reverse staining of nuclei. Finally, the sections were developed using the DAB detection kit (Zhongshan Biotech). The sections were evaluated by a pathologist who was blinded to the treatment group, and images were acquired using a Nikon SCLIPSS TE2000-S microscope (Nikon) with ACT-1 software. The original magnification was ×200.

### Statistical analyses

Data were presented as means and standard deviation (means ± SD). Statistical analyses were conducted with GraphPad Prism 8.2.1 Software. Statistical analysis of the comparison between the two groups adopted the two-tailed independent Student’s t-test. Multiple groups of comparison data were analyzed by one-way analysis of variance (ANOVA). The statistical *p*-value of different significance levels was set as *p < 0.05, **p < 0.01, ***p < 0.001, and ****p < 0.0001.

### Reporting summary

Further information on research design is available in the Nature Research Reporting Summary linked to this article.

### Supplementary information

Supplemental Material

REPORTING SUMMARY

## Data Availability

The data presented in this study are available on request from the corresponding author.
