# Supplementary material for: The co-delivery of adenovirus-based immune checkpoint vaccine elicits a potent anti-tumor effect in renal carcinoma
Source: NPJ Vaccines. 2023 Aug 4;8:109. doi: 10.1038/s41541-023-00706-x (PMC10403580; doi:10.1038/s41541-023-00706-x)
Supplement: Supplementary file 1 — Supplemental Material [file 41541_2023_706_MOESM1_ESM.pdf]

Supplementary Figures

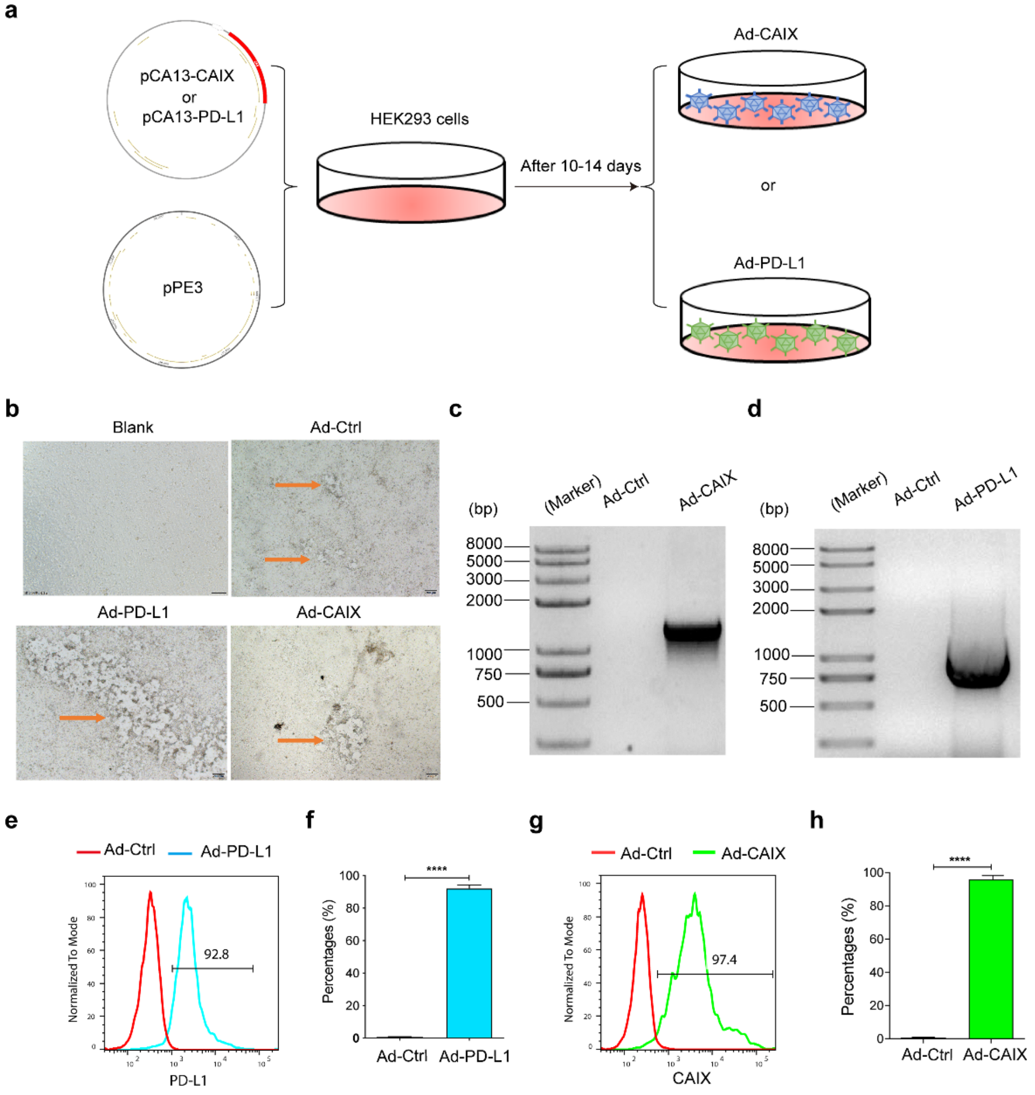

Supplementary Figure 1. The generation and expression of Ad-CAIX and Ad-PD-L1. a Schematic diagram of replication-deficient Ad packaging. **b** Plaque formation can be observed in Ad-Ctrl, Ad-PD-L1, and Ad-CAIX groups 14 days after co-transfection. **c, d** Identification of Ad-CAIX and Ad-PD-L1 by PCR analysis. HEK293 cells were respectively infected with Ad-Ctrl, Ad-CAIX, or Ad-PD-L1. The viral DNA extracted from the infected supernatant was used as DNA templates. Lane 1 displays DNA size standards (Kb). Lane 2 shows extracted Ad-Ctrl DNA as a template. Lane 3 shows extracted Ad-CAIX or Ad-PD-L1 DNA as a template. **e, f** The

high expression of Ad-PD-L1 in vitro. Purified Ad-PD-L1 are inoculated with HEK293 cells at MOI=5, respectively, while Ad-Ctrl is seeded at the same MOI as the control. The flow cytometry analysis was performed 48h after infection. **g, h** The expression of Ad-CAIX in vitro. Data were from one representative experiment of three performed and presented as mean ± SD. The different significance was set at ****p < 0.0001. Two groups of comparison data were analyzed by two-tailed independent Student's t-test.

Supplementary Figure 2

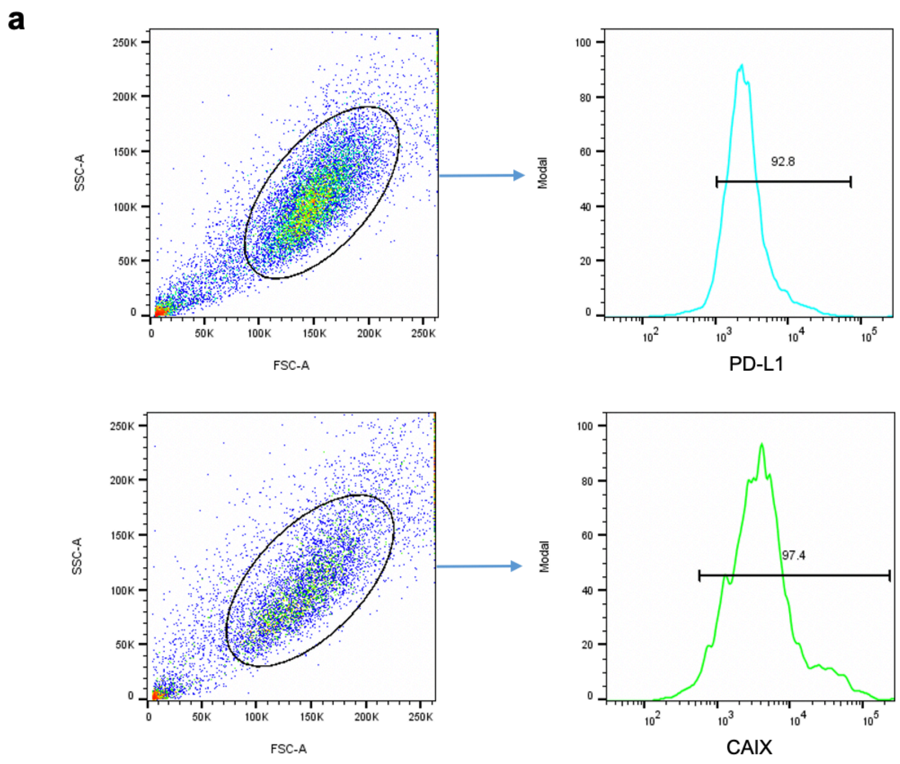

b

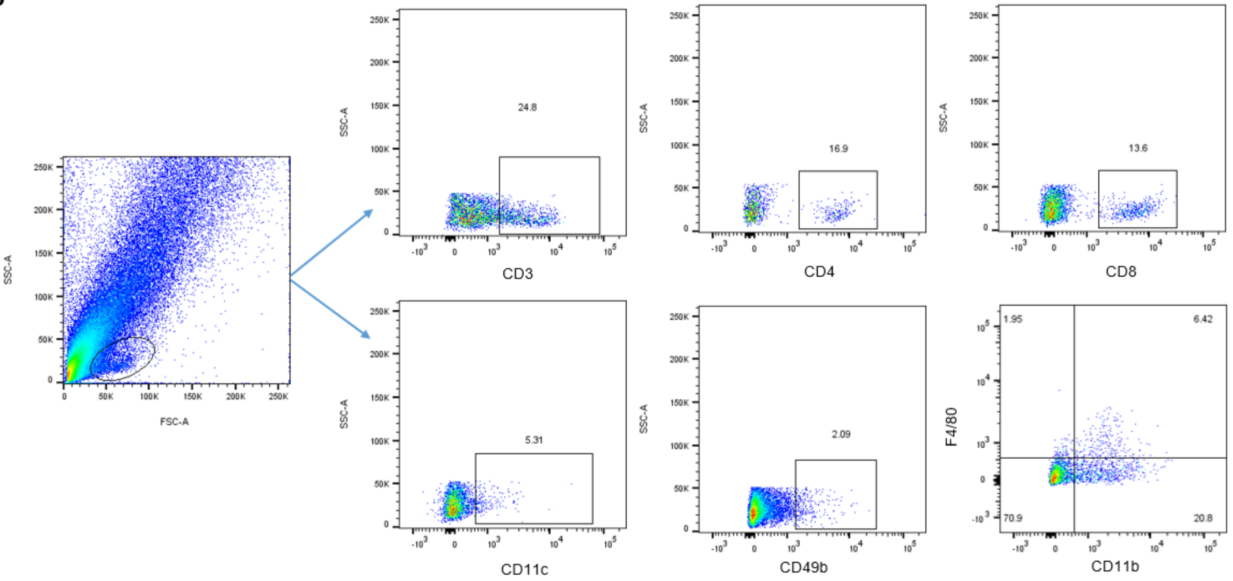
SSC-A
FSC-A
24.8
CD3
16.9
CD4
13.6
CD8
5.31
CD11c
2.09
CD49b
1.95
6.42
70.9
20.8
F4/80
CD11b

c

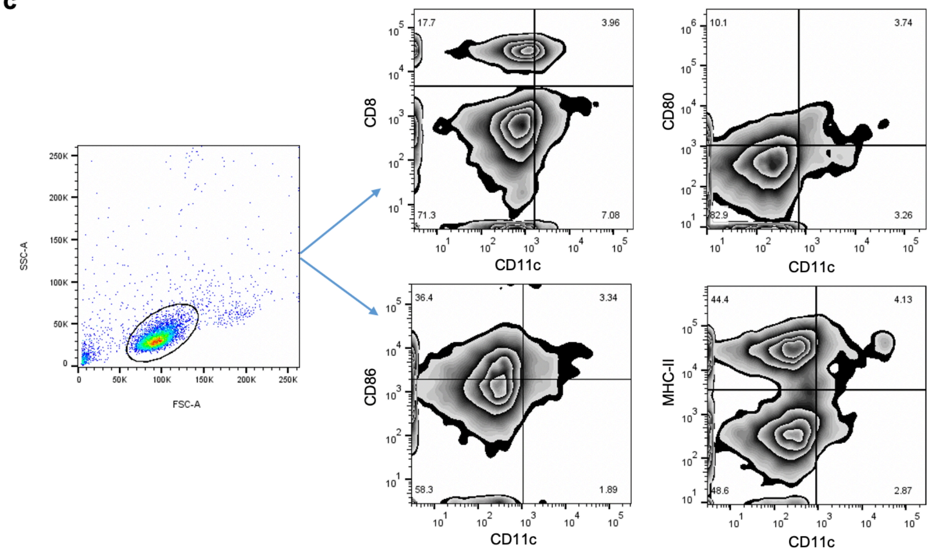
SSC-A
FSC-A
17.7
3.96
71.3
7.08
CD8
CD11c
10.1
3.74
82.9
3.26
CD80
CD11c
36.4
3.34
58.3
1.89
CD86
CD11c
44.4
4.13
48.6
2.87
MHC-II
CD11c

d

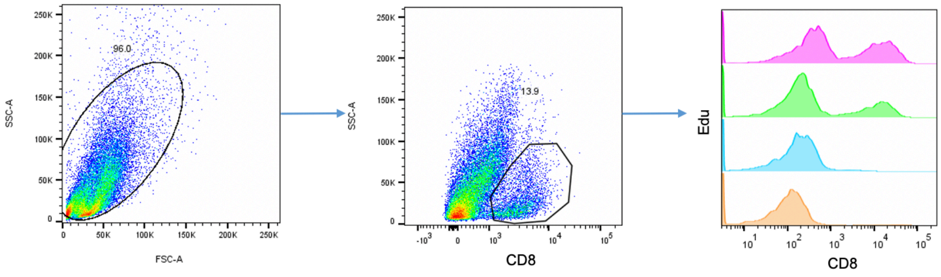
96.0
SSC-A
FSC-A
13.9
SSC-A
CD8
Edu
CD8

e

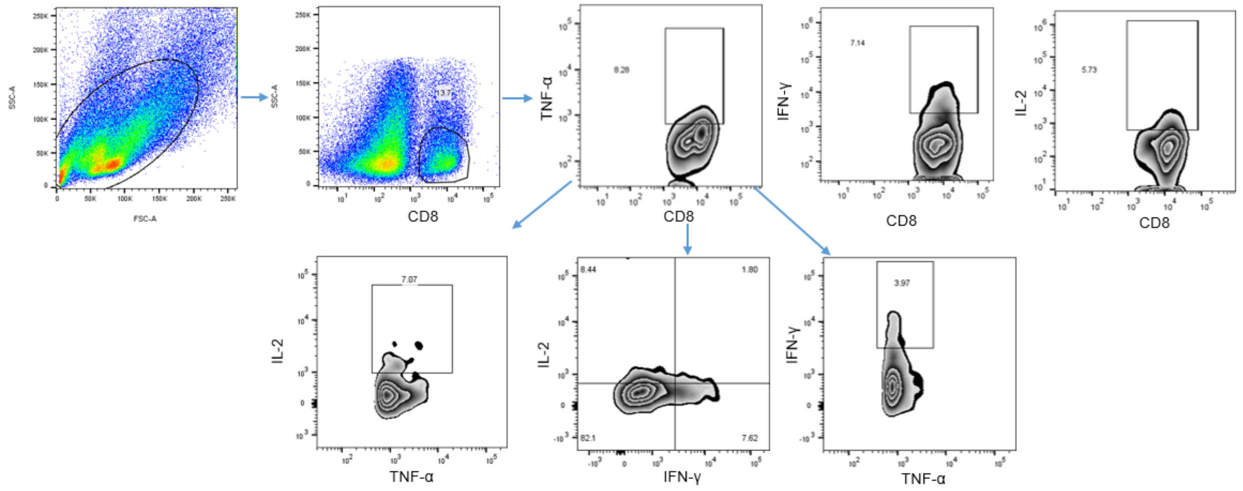

f

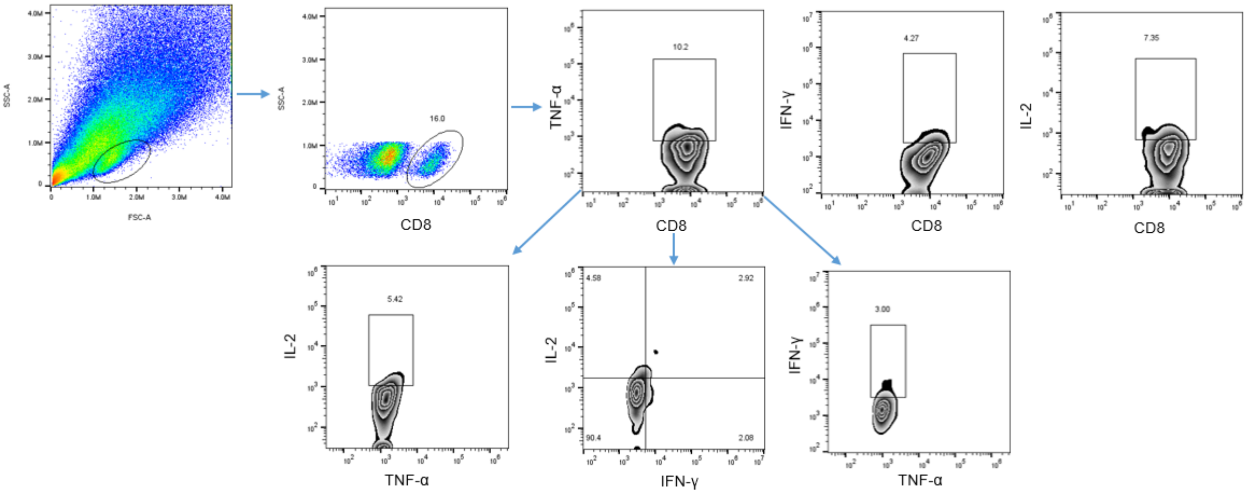

Supplementary Figure 2. Gating strategies used for cell analysis by flow cytometry. a Gating strategy to detect the expression of PD-L1 or CAIX on 293T cells infected by Ad-PD-L1 or Ad-CAIX presented on supplementary Fig. 1e, g. **b** Gating strategy to analyze the percentages of immune cells from tumors of immunized mice presented on Fig. 1e. **c** Gating strategy to analyze the percentages of $CD11c^{+}$ or $CD11c^{+}CD8^{+}$ cells and the expression of CD80, CD86, or MHC-II on $CD11c^{+}$ cells in the spleen of Ad vaccines-treated mice presented on Fig. 2a, d. **d** Gating strategy to analyze the percentages of $CD8^{+}Edu^{+}$ cells from the splenocytes of Ad vaccines-treated mice for in vitro cultures presented on Fig. 3a. **e, f** Gating strategy to analyze

the proportions of IL-2$^+$CD8$^+$, IFN-γ$^+$CD8$^+$, TNF-α$^+$CD8$^+$, TNF-α$^+$IFN-γ$^+$CD8$^+$, TNF-α$^+$IL-2$^+$CD8$^+$, IFN-γ$^+$IL-2$^+$CD8$^+$, and TNF-α$^+$IFN-γ$^+$IL-2$^+$CD8$^+$ T cells in splenocytes cultured in vitro or TILs from each group presented on Fig. 3d, Fig. 4a, b, Fig. 5g, and Fig. 6i, j.
