# Supplementary material for: The co-delivery of adenovirus-based immune checkpoint vaccine elicits a potent anti-tumor effect in renal carcinoma
Source: NPJ Vaccines. 2023 Aug 4;8:109. doi: 10.1038/s41541-023-00706-x (PMC10403580; doi:10.1038/s41541-023-00706-x)
Supplement: Supplementary file 2 — REPORTING SUMMARY [file 41541_2023_706_MOESM2_ESM.pdf]

nature portfolio

Corresponding author(s): Dafei Chai

Last updated by author(s): Dec 28, 2022

Reporting Summary

Nature Portfolio wishes to improve the reproducibility of the work that we publish. This form provides structure for consistency and transparency in reporting. For further information on Nature Portfolio policies, see our Editorial Policies and the Editorial Policy Checklist.

Statistics

For all statistical analyses, confirm that the following items are present in the figure legend, table legend, main text, or Methods section.

n/a	Confirmed	
☐	☒	The exact sample size (*n*) for each experimental group/condition, given as a discrete number and unit of measurement
☐	☒	A statement on whether measurements were taken from distinct samples or whether the same sample was measured repeatedly
☐	☒	The statistical test(s) used AND whether they are one- or two-sided<br>*Only common tests should be described solely by name; describe more complex techniques in the Methods section.*
☒	☐	A description of all covariates tested
☒	☐	A description of any assumptions or corrections, such as tests of normality and adjustment for multiple comparisons
☐	☒	A full description of the statistical parameters including central tendency (e.g. means) or other basic estimates (e.g. regression coefficient) AND variation (e.g. standard deviation) or associated estimates of uncertainty (e.g. confidence intervals)
☐	☒	For null hypothesis testing, the test statistic (e.g. *F*, *t*, *r*) with confidence intervals, effect sizes, degrees of freedom and *P* value noted<br>*Give P values as exact values whenever suitable.*
☒	☐	For Bayesian analysis, information on the choice of priors and Markov chain Monte Carlo settings
☒	☐	For hierarchical and complex designs, identification of the appropriate level for tests and full reporting of outcomes
☒	☐	Estimates of effect sizes (e.g. Cohen's *d*, Pearson's *r*), indicating how they were calculated

Our web collection on statistics for biologists contains articles on many of the points above.

Software and code

Policy information about availability of computer code

Data collection	N/A
Data analysis	Graph Pad Prism Version 8.2.1

For manuscripts utilizing custom algorithms or software that are central to the research but not yet described in published literature, software must be made available to editors and reviewers. We strongly encourage code deposition in a community repository (e.g. GitHub). See the Nature Portfolio guidelines for submitting code & software for further information.

Data

Policy information about availability of data

All manuscripts must include a data availability statement. This statement should provide the following information, where applicable:

- Accession codes, unique identifiers, or web links for publicly available datasets
- A description of any restrictions on data availability
- For clinical datasets or third party data, please ensure that the statement adheres to our policy

The data presented in this study are available on request from the corresponding author.

Human research participants

Policy information about studies involving human research participants and Sex and Gender in Research.

Reporting on sex and gender	N/A
Population characteristics	N/A
Recruitment	N/A
Ethics oversight	N/A

Note that full information on the approval of the study protocol must also be provided in the manuscript.

Field-specific reporting

Please select the one below that is the best fit for your research. If you are not sure, read the appropriate sections before making your selection.

☒ Life sciences ☐ Behavioural & social sciences ☐ Ecological, evolutionary & environmental sciences

For a reference copy of the document with all sections, see nature.com/documents/nr-reporting-summary-flat.pdf

Life sciences study design

All studies must disclose on these points even when the disclosure is negative.

Sample size	For animals studies, sample sizes were selected based on mean and SD observed in our vaccine studies with these animals models, and the ability to detect statistical differences between animal groups of this size. In vitro studies were performed with replicates and repeated at least three times.
Data exclusions	No data was excluded
Replication	All attempts at replication in the stated conditions were successful.
Randomization	For animal experiments, animals with the same gender, similar age and weight were randomly assigned to each group.
Blinding	N/A

Reporting for specific materials, systems and methods

We require information from authors about some types of materials, experimental systems and methods used in many studies. Here, indicate whether each material, system or method listed is relevant to your study. If you are not sure if a list item applies to your research, read the appropriate section before selecting a response.

Materials & experimental systems

n/a	Involved in the study	
☐	☒	Antibodies
☐	☒	Eukaryotic cell lines
☒	☐	Palaeontology and archaeology
☐	☒	Animals and other organisms
☒	☐	Clinical data
☒	☐	Dual use research of concern

Methods

n/a	Involved in the study	
☒	☐	ChIP-seq
☐	☒	Flow cytometry
☒	☐	MRI-based neuroimaging

Antibodies

Antibodies used	PE-conjugated anti-human CAIX (R&D Systems, Cat. FAB2188P), PE-conjugated anti-mouse CD3ε (BioLegend, Cat. 100308), APC-conjugated anti-mouse PD-L1 (BioLegend, Cat. 124312), APC-conjugated anti-mouse CD11c (BioLegend, Cat. 117310), PerCP-Cy5.5-conjugated anti-mouse CD4 (BioLegend, Cat. 116012), PerCP-Cy5.5-conjugated anti-mouse CD8α (BioLegend, Cat. 100734), PerCP-conjugated anti-mouse F4/80 (BioLegend, Cat. 123126), FITC-conjugated anti-mouse CD11b (BioLegend, Cat. 101206), APC-conjugated anti-mouse IFN-γ (Biolegend, Cat. 505810), FITC-conjugated anti-mouse TNF-α (Biolegend, Cat. 506304), and PE-

	conjugated anti-mouse IL-2 (Biolegend, Cat. 503808), Anti-mouse CD8α mAb (BioXCell, Cat. BE0004-1), Rat anti-mouse CD8 antibody (eBioscience, Cat. 14-0081-82), Goat anti-rat antibody (Zhongshan Biotech, Cat. PV9004).
Validation	See manufacturer's website for validation of commercial antibodies.

Eukaryotic cell lines

Policy information about cell lines and Sex and Gender in Research

Cell line source(s)	HEK293 cells: ATCC, CRL-1573; Renca cells: Cobioer Biosciences,CBP60451.
Authentication	The cell lines were not authenticated since they were purchased commercially and are not commonly misidentified.
Mycoplasma contamination	Cells tested negative for mycoplasma contamination.
Commonly misidentified lines (See ICLAC register)	N/A

Animals and other research organisms

Policy information about studies involving animals; ARRIVE guidelines recommended for reporting animal research, and Sex and Gender in Research

Laboratory animals	Mice were purchased from the Laboratory Animal Center of Xuzhou Medical University (Xuzhou, China) and housed under specific-pathogen-free (SPF) conditions with standard temperature and humidity.
Wild animals	N/A
Reporting on sex	N/A
Field-collected samples	N/A
Ethics oversight	All the animal procedures and protocols were authorized by the Laboratory Animal Ethical Committee of Xuzhou Medical University. All performances accord with the guidelines for the Care and Use of Laboratory Animals of Xuzhou Medical University.

Note that full information on the approval of the study protocol must also be provided in the manuscript.

Flow Cytometry

Plots

Confirm that:

- [x] The axis labels state the marker and fluorochrome used (e.g. CD4-FITC).
- [x] The axis scales are clearly visible. Include numbers along axes only for bottom left plot of group (a 'group' is an analysis of identical markers).
- [x] All plots are contour plots with outliers or pseudocolor plots.
- [x] A numerical value for number of cells or percentage (with statistics) is provided.

Methodology

Sample preparation	Cell line or the single-cell suspension of spleens and tumor tissues were prepared as described in the methods section.
Instrument	Data were collected on a BD FACSCanto II instrument as stated in the methods section.
Software	Data were analyzed in FACSDiva and FlowJo software as stated in the methods section.
Cell population abundance	N/A
Gating strategy	The gating strategy is shown in Figures of the manuscript.

- [ ] Tick this box to confirm that a figure exemplifying the gating strategy is provided in the Supplementary Information.
